# Whole-Blood Gene Expression Profiles in Large-Scale Epidemiological Studies: What Do They Tell?

**DOI:** 10.1007/s13668-015-0143-5

**Published:** 2015-10-08

**Authors:** Karina Standahl Olsen, Guri Skeie, Eiliv Lund

**Affiliations:** Department of Community Medicine, UiT Arctic University of Tromsø, 9037 Tromsø, Norway

**Keywords:** Nutrigenomics, Gene expression, Transcriptomics, Blood, PBMC, Nutrition, Epidemiology, Diet, Nutrients, Food, Dietary patterns, Immunology, Inflammation, Study design, Intervention, Cohort, RCT, mRNA, Whole blood, Prevention

## Abstract

In nutrigenomics, gene expression profiling is used to investigate transcriptional mechanisms associated with nutrients and diet. Blood samples collected in the framework of dietary interventions and epidemiological studies allow the use of humans as the model system, as opposed to using cell lines or animal models. Here, we review recent publications in the field of gene expression profiling, based on a systematic literature search focusing on studies from the last 5 years and including studies that investigated either single nutrients, foods, food groups, or dietary patterns. Findings highlight the role of inflammatory processes as key mediators of the association between diet and disease and point to the relevance of using blood as the target tissue in nutrigenomics. However, recurring challenges include study design issues, practical and statistical challenges, and biological interpretation of the results. Many of the published studies have small sample size, and given the nature of gene expression data, their conclusions have limited impact. These challenges should be addressed by future nutrigenomics studies in order to increase their relevance and validity.

## Introduction

High-throughput omics technology is increasingly being used to elucidate the complex interactions between exposures such as diet and pollutants and human health and disease. In the nutrigenomics field, the analysis of messenger RNA (mRNA) expression, known as gene expression profiling or transcriptomics, allows a genome-wide perspective on the molecular mechanisms involved in the association between nutrients, foods, dietary patterns, and health outcomes. Numerous dietary components have been identified as signaling molecules that may alter gene expression and thereby influence health [1]. Transcription factors are the main mediators through which nutrients influence gene expression (e.g., peroxisome proliferator-activated receptors that bind fatty acids [2] and electrophile response elements activated by plant phytochemicals [3]), but numerous other and/or more indirect mechanisms may be just as influential [4••]. Recently, a review of microRNAs concluded that these nucleic acids also play a role in dietary modulation of health and disease [5]. An overview of transcription factors and other mechanisms is beyond the scope of this review.

There is a growing understanding of the limitations of using animal models and cell lines to explore human pathogenic processes [6••]. The degree of match between transcriptomic inflammatory responses in humans and mice was found to be close to random [7], highlighting the need for more studies using humans as the model system. Studies of blood cells are particularly useful for combining epidemiological and molecular aspects of biomedical research/nutrigenomics, as blood sample collection is relatively non-invasive and feasible in the epidemiological setting. Being a part of the transport system of the body, blood cells interact with all tissues and are exposed to nutrients, metabolites, excreted factors, and waste products. Hence, blood cells have been suggested as a surrogate tissue for the study of molecular responses to dietary interventions [8] and of disease processes in other tissues [9]. However, the transcriptionally active blood cells are key immunological regulators and are in themselves highly relevant as a target tissue for elucidating the impact of diet and nutrition on health and disease [10••].

The immune system has evolved to protect us from external challenges, but it also plays a vital role in maintaining homeostasis. Diseases with a substantial immunological aspect include cancer, cardiovascular disease (CVD), and diabetes, and the pathogenesis and incidence of these diseases are also affected by diet [11]. Cells of both the innate and adaptive immune system are present in the blood, at varying numbers and varying developmental stages, and the white blood cells (leukocytes, monocytes, granulocytes) are the most transcriptionally active, in contrast to the red blood cells (reticulocytes) and platelets. Advances in gene expression analysis and blood sample handling have given the opportunity to assess transcriptional effects of diet in blood cells [9]; however, a rich body of data from other methodologies has provided several clues to the link between inflammation and diet. These include regulation of levels of cytokines, production of signaling molecules like eicosanoids and resolvins, oxidative stress, DNA damage, and viability of immune cells [12]. Furthermore, inflammatory processes at the physiological level may include vascular wall flexibility, lipid metabolism, and systemic low-grade inflammation which has been linked to obesity and lifestyle-related diseases [13, 14].

In this review, we will discuss recent publications (Table 1) that present analyses of whole-blood gene expression profiles related to single nutrients, foods/food groups, and dietary patterns. We identify trends within the field and discuss several challenges that should be met by future nutrigenomics studies in order to increase the validity of reported findings.

**Table 1:** Recent whole-blood gene expression profiling studies examining effects of nutrients, foods, food groups, and dietary patterns

Author	Year	Country	Design	Study population	Exposure	Group *n* ^a^	Time of sampling	Main results
Bouchard-Mercier [15]	2013	Canada	Cross-sectional	Healthy	Western and prudent dietary patterns	3 to 9	–	Both prudent and Western pattern influenced GE; few transcripts overlapped in men and women.
Bøhn [16]	2010	Norway	RCT, repeated measurements	Male smokers	Kiwi or anti-oxidant diet	9 or 10	0 and 8 weeks	Increased defense responses in both intervention groups.
Castaner [17]	2013	Spain	RCT, repeated measurements	At risk of CVD	Mediterranean diet with olive oil or nuts	11	0 and 3 months	Mediterranean diet with olive oil supplementation modulated GE of cardiovascular risk genes.
Choi [18]	2012	Korea	Intervention, time series	Healthy	Glucose	60	0, 1, and 2 h	Modulation of NK cell-mediated immunity, granulocyte-mediated immunity, and the cytokine-mediated signaling pathway.
Ellsworth [19]	2014	USA	Non-randomized controlled intervention, time series	At risk of CVD	Fat reduction and physical activity	63	0, 12, and 52 weeks	Reduced GE of neutrophil activation genes, as well as cytokine production, carbohydrate metabolism, and steroid hormone pathways.
Hochstenback [20]	2012	Norway	Cross-sectional, dose-response	Neonates, cord blood	Maternal TCDD, PCB	45 and 66	–	Suggestive immunosuppressive effect. Genes correlating negatively with exposure in general showed positive correlations with antibody levels.
Kawakami [21]	2013	Japan	Intervention, cross-over	Healthy	Glucose, white rice, barley	7	0 and 6 h	Differential GE of glycolysis and fatty acid beta-oxidation genes.
Marlow [22]	2013	New Zealand	Intervention, repeated measurements	Crohn’s patients	Mediterranean diet	8	0 and 6 weeks	Processes including EIF2 signaling, B cell development, and Th cell differentiation.
Milenkovic [23]	2011	France	RCT cross-over	Overweight males	Orange juice or hesperidin	10	4 weeks	Common processes in the two intervention groups: chemotaxis, adhesion, infiltration, and lipid transport.
Milenkovic [24]	2014	France	Intervention, repeated measurements	Healthy male smokers	Flavanols from grape seeds	7	0 and 8 weeks	Genes suggestive of lower immune cell adhesion to endothelial cells.
Myhrstad [25]	2014	Norway	RCT, repeated measurements	Healthy	Fish oil	18	0 and 7 weeks	Differential GE of cell cycle, endoplasmic reticulum stress, and apoptosis genes.
Olsen [26]	2013	Norway	Cross-sectional	Healthy	PUFA ratios: n-6/n-3, LA/ALA, AA/EPA	23	–	Tendency for increased pro-inflammatory signaling and lower autophagy GE in the highest PUFA ratio groups.
Olsen [27]	2013	Norway	Cross-sectional	Healthy	Vitamin D (25(OH)D)	66 to 83	–	Modest effects. Immunological processes, immune cell functions, and major signaling pathways.
Rudkowska [28]	2011	Canada	Intervention, repeated measurements	Obese	n-3 PUFA and fish gelatin	14 to 16	0 and 8 weeks	Distinct but comparable effects of the two interventions. PPAR-α and inflammatory pathways.
Rudkowska [29]	2013	Canada	Intervention, repeated measurements	Healthy	n-3 PUFA	29	0 and 6 weeks	Different magnitude of effects in men and women. PPAR-α, NF-κB, and oxidative stress pathways.
Ryu [30]	2011	USA	Intervention, time series	Healthy males	Zink depletion	9	0, 6, and 10 days	Cell cycle regulation and immunity processes.
Sagaya [31]	2012	Switzerland	RCT cross-over, time series	Healthy males	Milk and yogurt	6	0, 2, 4, and 6 h	Similar processes affected by the two interventions. Protein biosynthesis, mitochondrial activities, inflammatory and apoptotic processes.
Schmidt [32]	2012	Germany	RCT, time series	Healthy and dyslipidemic	Fish oil	6 to 9	0, 4 h, 1 week, and 12 weeks	Larger effect in dyslipidemics with several pro-inflammatory genes down-regulated.
Schmidt [33]	2012	Germany	Intervention, time series	Healthy and dyslipidemic	Fish oil	7 and 9	0, 4 h, 1 week, and 12 weeks	Results suggestive of anti-oxidative and potentially cardioprotective effects of fish oil.
Tome-Carneiro [34]	2013	Spain	RCT, time series	Hypertensive T2D males	Grape extract and resveratrol	6	0, 6, and 12 months	Down-regulation of GE of key pro-inflammatory cytokines and related miRNAs.
van Breda [35•]	2014	Netherlands	Intervention, repeated measurements	Healthy	Blueberry-apple juice	56 to 143	0, 4 weeks	Modulation of apoptosis, induced immunity, reduced platelet aggregation and activation, blood glucose homeostasis, and regulation of fatty acid metabolism.
van der Velpen [36]	2013	Netherlands	RCT cross-over	Healthy, postmenopausal women	Isoflavone	28	8 weeks	Down-regulation of inflammation, oxidative phosphorylation, and cell cycle. No significant effect on estrogen receptor target genes.
van Dijk [37]	2012	Netherlands	Controlled intervention, repeated measurements	Overweight	Mediterranean diet and MUFA diet	15 and 17	0 and 8 weeks	Reduced metabolic stress and oxidative phosphorylation. The Mediterranean diet may have additional anti-atherogenic effects.
Vedin [38]	2012	Sweden	RCT	Alzheimer patients	n-3 PUFA	11 and 5	6 months	Modulation of inflammatory processes relevant to Alzheimer’s disease.

^a^Group *n* denotes the number of samples in each comparison group with successfully obtained gene expression profiles if this number was stated in the publication. Otherwise, and for cross-over studies, the number of included subjects is given. *AA* arachidonic acid, *ALA* alpha-linolenic acid, *CVD* cardiovascular disease, *EPA* eicosapentaenoic acid, *GE* gene expression, *LA* linoleic acid, *NF-κB* nuclear transcription factor kappa B, *PCB* polychlorinated biphenyl, *PUFA* polyunsaturated fatty acids, *PPAR-α* peroxisome proliferator-activated receptor alpha, *RCT* randomized, controlled trial, *T2D* type 2 diabetes mellitus, *TCDD* dioxin

## Gene Expression Profiles Associated with Intake of Specific Nutrients

Due to their putative effects on CVD risk factors, incidence, and death [39, 40], as well as risk of cancer [41] and type 2 diabetes mellitus [39], dietary fat has spurred several investigations. Fatty acids take part in cellular processes including gene expression modulation via nuclear receptors, acting as precursors for inflammatory signaling molecules, and are also structural components of cells [42, 43]. Two recent reviews summarized the reported effects of marine n-3 FAs on peripheral blood mononuclear cell gene expression [44] and the potential use of blood transcriptomics as biomarkers for fatty acid intake [45••]. Briefly, supplementation trials have been carried out in populations of varying health status, mainly healthy subjects or subjects with lifestyle-related conditions such as obesity, insulin resistance, or dyslipidemia. Studies often include <30 persons in each comparison group, and the duration of interventions ranges from a few weeks to 6 months. Studies using full-genome approaches have revealed approximately 200–1000 differentially expressed genes, collectively giving an indication of the magnitude of the transcriptional impact of n-3 polyunsaturated fatty acid (PUFA) supplementation. Importantly, differences between population strata have been shown. Men [29] and dyslipidemic subjects [32] may be more affected by supplementation compared to women and subjects with a normal lipid profile. The abovementioned review papers conclude that transcriptomic biomarkers are indeed sensitive to dietary interventions using fatty acids and that affected mechanisms include pro- and anti-inflammatory processes, anti-atherogenic pathways, and fatty acid metabolism. One recent study by Myhrstad et al. found that genes related to endoplasmic reticulum stress may also be linked to an increased oxidative load on cells after PUFA supplementation [25]. Lastly, transcriptomic effects of fatty acid ratios have been shown in a cross-sectional study of women in a general population [26], indicating that differences in blood fatty acid composition among persons following their habitual diet may affect the same molecular mechanisms usually documented in controlled trials.

Due to their possible roles in oxidative processes [46] relevant for both inflammation and tissue homeostasis, molecular effects of some plant-derived compounds have been explored. Epidemiological data indicate that consumption of fruits and vegetables rich in phytochemicals may reduce risk of CVD [47] and some cancers [48], but only a few health effects have been determined for isolated compounds. Afman et al. [45••] reviewed and discussed recent transcriptomic supplementation studies including 7–30 subjects who were healthy or at risk of CVD. Comparison of gene lists from trials investigating quercetin, resveratrol, hesperidin, and isoflavones revealed effects on several common pathways: chemokine signaling, cell adhesion, apoptosis, and NF-κB, all involved in inflammation and atherogenesis [45••]. Recently, in a study of seven healthy male smokers, flavanol supplementation was found to induce 864 genes related to chemotaxis, cell adhesion, and intracellular processes such as cytoskeleton organization [24]. The authors suggest that flavanols may lower immune cell adhesion, with favorable impact on CVD pathogenesis, in line with Afman’s conclusions [45••]. Down-regulation of inflammatory processes was also found in a randomized controlled trial of isoflavones in postmenopausal women. However, the potentially harmful effects on estrogen receptor pathways were not found in the blood of these women [36]. Studies of fruit/berry juices are discussed below.

Not many studies have focused on vitamins and minerals/trace elements. Vitamin D status was not associated with any significantly differentially expressed single genes in a cross-sectional analysis of 149 postmenopausal women [27]. However, at the pathway level, immunological processes were associated with higher vitamin D status [27]. After zinc depletion in nine male subjects, 328 genes were differentially expressed, with cell cycle regulation being up-regulated and immune response being down-regulated [30]. In contrast to the vitamin D study [27], the results on zinc depletion were found to align with previously reported in vitro findings.

Two studies of 7 and 60 subjects investigated acute effects of carbohydrate ingestion. After 1 h, glucose ingested by 60 healthy subjects induced differential expression of 36 genes related to NK cell and granulocyte immunity, as well as cytokines [18]. The authors pointed out that the observed effects could also be a result of secondary systemic changes, like hyperinsulinemia resulting from the glucose ingestion. In a cross-over study of seven subjects, meals with differing glycemic indexes (provided by glucose, white rice, and rolled barley) were associated with distinct gene expression profiles 6 h after ingestion [21].

## Gene Expression Profiles Associated with Foods and Food Groups

Juices and extracts of fruits and berries have been studied in trials including 9–143 persons. The largest study examined effects of 1 l of blueberry juice per day for 4 weeks [35•], and the authors report 1500–9000 differentially expressed genes (including non-annotated genes), belonging to processes like apoptosis, immune response, cell adhesion, and lipid metabolism. Interestingly, response varied according to genotype subgroups, and according to subgroups that were defined by degree of DNA damage. In a 1-year placebo-controlled, triple-blinded trial including hypertensive males with type 2 diabetes and coronary artery disease, grape extract fortified with resveratrol down-regulated key inflammatory regulators (TNF-α, IL-1b, IL-8), indicating an attenuation of NF-κB-signaling [34]. Orange juice was found to alter the expression of 3422 genes after 4 weeks in ten subjects, and some of this effect was linked to the flavanone hesperidin [23]. The identified differentially expressed genes contribute to inflammatory regulation by modulating endothelium interaction (cell adhesion and infiltration) and lipid accumulation, altogether suggested as a cardioprotective expression profile. Finally, a study of male smokers identified several stress defense genes up-regulated after an 8-week dietary intervention with either a combination of anti-oxidant-rich plant foods or a kiwi fruit supplementation compared to controls following their habitual diet [16]. The anti-oxidant-rich diet resulted in 44 differentially expressed genes, and the kiwi supplementation group nine genes were differentially expressed. Gene set analysis identified the up-regulation of several stress-related gene sets, particularly DNA and repair, hypoxia, and apoptosis in both intervention groups [16]. Health benefits related to anti-oxidant-rich diets may therefore be linked to up-regulation of stress and defense responses to maintain cellular repair processes.

One study investigated the acute transcriptomic effect of ingestion of milk and yogurt in six subjects, identifying around 600 differentially expressed genes after 6 h [31]. Although only 11 % of the genes were overlapping in the two groups, pathway-level analyses revealed similar processes including protein biosynthesis and mitochondrial activities (up-regulated) and inflammation and apoptosis (down-regulated). The authors point to the transcriptional kinetics shortly after ingestion as a relevant aspect of nutrition in relation to prevention and development of diseases [31].

Intake of fatty foods, like fish, olive oil, etc., has not been extensively studied per se. Fish oil supplementation was discussed in the previous section, as these studies focus on the fatty acid content, and possible effects of olive oil have been investigated in relation to the Mediterranean dietary pattern (next section). Also, intake of grains has been evaluated as part of dietary patterns, and one study of differing effects of meals with varying glycemic indices was included in the previous section.

## Gene Expression Profiles Associated with Dietary Patterns

In the real-life setting, nutrients are provided as part of complex dietary patterns, not as single, isolated entities, and the WCRF/AICR recommends that healthy persons aim at meeting nutritional needs through diet alone, as opposed to using supplements [48]. This more global view of nutrition has also been investigated using transcriptomics, particularly focusing on the Mediterranean diet as previously summarized by Konstantinidou [49•]. Recent studies include transcriptomic data from 3 to 17 persons in each comparison group, and in line with epidemiological findings, the Mediterranean dietary pattern has been shown to reduce expression of genes related to inflammation (e.g., IL-1b, TNF-α, ICAM, NF-κB) and oxidative phosphorylation/oxidative stress/hypoxia (e.g., HIF1a, VEGF), possibly owing to the high contents of MUFA, PUFA, and anti-oxidants [17, 37]. However, findings are not always consistent [37] and the positive effects may be increased by further enriching the diet with olive oil [17]. The affected processes are linked to CVD development, and altering the transcriptome as a “molecular symptom” of disease may be one way that diet helps to prevent onset and progression of CVD and other inflammatory diseases. Similar trends were found in Crohn’s patients [22]. The Mediterranean diet is often contrasted to a Western dietary pattern, which was studied in a cross-sectional analysis of 30 persons following their habitual diet [15]. Subjects with a Western-type diet had higher blood pressure and gene expression profiles indicative of higher inflammatory status. Importantly, this study identified major differences in gene expression profiles in men and women [15]. Other systemic approaches, like fat reduction in combination with exercise, have been investigated. In 63 CVD patients and equal number of matched controls, a strict dietary and lifestyle intervention led to alterations of genes related to leukocyte function, lipid homeostasis, and inflammation [19]. Interestingly, adverse effects after maternal immunotoxic exposure to PCB and dioxins derived from seafood, dairy, egg, and cereals were associated with an immunosuppressive gene expression profile in cord blood, as well as reduced vaccine response in offspring [20]. This study highlights the potential of using transcriptomics to investigate vulnerable population strata and to explore transgenerational aspects of health.

## Discussion

Some overall trends may be identified in the available publications of whole-blood gene expression profiling in the field of nutrigenomics. Firstly, there is a trend toward using the blood as the target tissue, instead of it being a surrogate for other tissues. In line with trends in cancer research [50, 51•] and other fields [14], the involvement of the immune system in disease etiology and prevention is increasingly being recognized. The published gene expression profiles discussed here repeatedly point to the immune-modulatory effects of the diet, and this deserves further attention. What is the role and impact of these effects at the population level, what other factors besides nutrients may affect the same mechanisms, and how might these effects be targeted in different population strata to improve public health? The magnitude of immunological consequences of diet, both positive or negative, remains to be established, but transcriptomic studies as well as epidemiological data from research on cancer, CVD, and obesity point to its relevance. Whole-blood gene expression profiles may reflect systemic changes to a larger degree than expression profiles derived from adipose tissue or other tissues, and investigations of both local and systemic effects of nutrients and diet will help to elucidate the complex mechanisms associated with dietary components. However, the understanding of the role of adipose tissue in inflammatory and pathogenic processes has increased during the last 5 years. For example, a recent study demonstrated transcriptomic changes in abdominal subcutaneous adipose tissue (SAT) of obese persons after an 18–24-week dietary intervention using the Nordic diet compared to a control diet [52]. The Nordic diet, in absence of weight change, was associated with decreased expression of inflammatory genes in SAT, which might influence systemic disease processes through reduced secretion of inflammatory mediators from the adipose tissue. However, as the expression profiles were not associated with major changes in plasma concentrations of inflammatory markers or insulin sensitivity, the clinical impact of the reported findings in SAT remains to be established [52]. It should be mentioned that it may be more difficult to reach adequate sample size in studies that require tissue biopsies compared to studies using blood samples.

Secondly, the field of whole-blood transcriptomics in nutrition research bears similarities to the early days of genome-wide association studies of single-nucleotide polymorphisms (SNPs), where discoveries and claims were abundant [53]. The technology is costly which leads to small sample sizes and a high risk of false positives. Of note, some (but not enough) studies are indeed presented as pilot or feasibility studies, with the need of future replication. The challenge of small sample sizes is further discussed below. The amount of data available today, especially for whole-blood gene expression profiles in response to fatty acid intake, is eligible for meta-analyses in spite of differences in microarray platforms, study designs, and target populations. Meta-analyses could reveal canonical molecular processes at play at the population level. One example of this approach was provided in [45••] for polyphenols, leading to a hypothesis of synergistic effects of a variety of polyphenol-rich plant foods on inflammation and atherogenesis.

Lastly, many challenges adhere to the analysis of blood gene expression profiles in nutritional epidemiological studies. These are discussed below and include study design issues, as well as practical and statistical challenges, and biological interpretation of results. Being a multidisciplinary field, the standards of all involved disciplines must be met in order to increase the quality of the published studies and the validity of reported findings.

### Challenges

In nutrigenomics, estimation of sample size to reach adequate statistical power may not be straightforward, due to lack of prior information on effect size and variability. The magnitude of the expected findings is often low: the impact of an exposure (say, a single nutrient) may not stand out from the impact of a myriad of other exposures present at the sampling point. This raises a demand for rigorous study design and recording of potential confounding factors, and stringent study designs may not always provide confirmation of initial results, as was the case with one study of quercetin [54]. Importantly, the majority of studies discussed herein include below 20 subjects in each comparison group, so the term “large-scale epidemiological studies” as stated in the title of this review may well be discussed. Increased sample sizes will improve the precision of the estimates, lower the risk of confounding, and allow stratification into subgroups to further reveal the complexity of gene/lifestyle/diet interactions, as was done by van Breda et al. [35•]. Several published studies base their choice of sample size on an argument that other studies with the chosen sample size have been able to identify gene expression effects of diet. However, with knowledge of the methodological challenges of gene expression profiling, it is evident that this reasoning falls short: gene expression profiling generates “noisy data” (low signal to noise ratio), is prone to batch effects, and is subject to the heterogeneity of the biological samples used. This leads to low reproducibility in independent datasets and difficulty in establishing global signatures of the exposure in question [55••]. In summary, more studies with adequate sample sizes are needed in order to capture the signals that are truly associated with the exposure of interest.

Furthermore, recording non-dietary lifestyle factors and including biomarker measurements and other omics technologies (genomics, epigenomics, proteomics, metabolomics) in the study designs may provide important clues to between-person variability of response to nutrients, help in biological interpretations, and may inform nutrition intervention practice [55••, 56]. The use of healthy subjects will increase our appreciation of diet in disease prevention, but possible effects in persons at risk of disease or with different diagnoses may provide clues for recommendations of lifestyle changes for patients. In addition, examining effects in vulnerable population strata, such as children and youth, may further strengthen the efforts to improve dietary habits early, with the aim of life-long benefit. Similar to initiatives in the field of genome-wide association studies, consortium-based approaches may help overcome the problem of small sample sizes and provide large enough sample sizes to allow stratification and increase statistical power [57••].

Careful study planning including the use of washout periods, choice of nutritionally relevant doses, and the use of (and choice of) placebo treatment may strengthen future intervention studies. In cohorts, recording of dietary data together with blood sample collection may prove to be valuable for avoiding many of the potential biases of case-control studies. Repeated measurements in cohorts may provide insight into the temporal relation of diet and disease development. Also, exploring transcriptomics as biomarkers of exposure or early biological effects is promising [45••].

Practical challenges related to conduct of transcriptomic analyses have been discussed elsewhere and include stabilization of mRNA [58–60], globin reduction methods [61, 62], batch effects [63], and recognition of between- and within-person variability [64]. We previously found that laboratory procedures may account for up to 40 % of gene expression variance [65], and today, evidence-based guidelines for pre-analytical handling of blood samples are available and may increase validity and reproducibility [58]. Furthermore, in matched designs, sample pairs should be kept together throughout the analysis pipeline (both wet lab and data analysis) in order to minimize the impact of technical variability. Data analysis of transcriptomics is a multistep process involving quality control, outlier detection, normalization, log transformation, and detection of differentially expressed genes and pathways [55••, 66]. The non-hypothesis-driven approach allows researchers to explore the data in an agnostic fashion, but best-practices are yet to be established. Performing pathway-level analyses and using network-based approaches have proven to be a vital asset for biological interpretation of transcriptomic datasets [67•], and these approaches are particularly useful in the nutrigenomics setting where many exposures may induce non-significant changes at the single-gene level.

The available literature not only provides a link between previously published epidemiological findings and in vitro studies, but also points to molecular mechanisms with unknown links to health effects at the population levels. One example is that of endoplasmic reticulum stress and authophagy found to be associated with higher n-3 PUFA both in the general population and after supplementation [25, 26]. However, most studies are not able to distinguish between direct transcriptomics effects of the exposure in question and the transcriptomic effects of secondary systemic changes resulting from the exposure. As already pointed out, the use of established biomarkers in conjunction with transcriptomics may help biological interpretation, but transcriptomic data may not always reflect changes of inflammatory protein markers such as TNF-α [34]. This points to the fact that mRNA is only one piece of the biological puzzle: posttranscriptional and posttranslational modifications and other physiological processes will affect both the amount of markers in the circulation as well as the physiological impact of diet. Transcriptomics as such increases the analytical depth and contributes to our understanding of biological complexity.

## Conclusion

We conclude that the published literature is characteristic of a new research field where sample sizes are small and reported findings may not always be confirmed by larger and more well-designed studies. Still, transcriptomic data is accumulating that contributes to the understanding of the processes underlying the health impact of diet. The reported findings help to combine and confirm in vitro and epidemiological data and point to new associations that deserve the attention of future studies. However, based on the available literature, few conclusions about etiological associations can be made. Gene expression studies are intrinsically hypothesis generating, and complementary approaches are needed to further explore the molecular mechanisms at play and their impact at the population level. However, for the hypotheses to be worth exploring, the studies that generate them must be carefully designed and meet the high standards of research in both epidemiology, nutrition, and molecular biology. Based on the rationales discussed herein, it is evident that studies exploring transcriptomic effects of nutrients, foods, and dietary patterns in large-scale epidemiological studies are highly warranted.
